# Supplementary material for: Acceptance of COVID-19 vaccination in a sample of Middle Eastern and North African (MENA) Houston residents
Source: PLOS Glob Public Health. 2023 Feb 1;3(2):e0001441. doi: 10.1371/journal.pgph.0001441 (PMC10021233; doi:10.1371/journal.pgph.0001441)
Supplement: Attachment — Submitted filename: Response Letter.docx [file pgph.0001441.s001.docx]

**RESPONSE LETTER**

We are grateful to the editor and the reviewers for a careful review of our manuscript and appreciate the opportunity to resubmit our work to the journal. Some concerns were raised which we have addressed in the order raised. We have further strengthened our study by including pertinent discussion and made edits as needed which can be viewed under track change feature.

**Reviewer #1**: The authors have done stellar work in this paper. I offer a few suggestions meant to strengthen the manuscript:

**1.** The introduction is very long and can be shortened. Consider starting with a brief overview of the research that has been published about Covid-19 vaccination hesitancy and how this research has focused on minorities and whites. Then transition to the fact that MENA are identified as white. The section about misinformation can be moved into the discussion section. You should retain the data about MENA in Texas. The paragraph on page 5 (lines 104-113) can be integrated to the discussion section.
**Response:** Thank you for your kind comments and for providing excellent suggestions on improving the introduction section. Following reviewer suggestions, we have now revised the introduction section as recommended.

**2.** On page 6, under the Survey section, you do not need to name the person who developed the survey. I would suggest you write something like "one of the co-authors."
**Response:** Excellent point. Suggested edit has been incorporated as recommended.

**3.** On page 7, line 144 needs a reference after you mention the survey that was developed. I am curious: were some of the survey questions borrowed from other surveys about Covid-19 vaccine hesitancy? Either way, this should be stated.
**Response:** The survey was originally conducted on a diverse sample of American women with different racial/ethnic groups (<https://doi.org/10.1093/tbm/ibab014>). The survey was then translated in Arabic and used for a national survey conducted in Arab Americans (<https://doi.org/10.1007/s40615-021-01158-6>).

**4.** When describing the tables, I suggest the authors highlight a few of the findings. There is no need to describe each finding, especially in tables 1 and 2.
**Response:** Done as recommended.

**5.** In the multivariable logistic regression model, I suggest the authors present a crude, unadjusted odds ratio and confidence intervals and then add variables based on grouping, such as demographics, health behaviors, health conditions, Covid-19 characteristics. I also suggest the authors present a main independent variable versus including all of the covariates to see which ones have statistically significant influence. For example, based on previous research and knowledge about the MENA community, there may be a reason to believe that Covid-19 characteristics (infected, risk, severity, self-protection, avoidance) are the main predictors of vaccine hesitancy. So, the authors would present crude odds ratios for those (or even create a score of the Covid-19 characteristics) and then add "groupings" of demographic, health behaviors, etc., that may influence the crude estimates. I think this presentation will make the analysis more methodical and justified.
**Response:** As suggested, we have added the unadjusted ORs and associated p values to the revised table. This is a hypothesis generating cross sectional study which was conducted to identify which variables affect the outcome (vaccination). Our plan for future work is to build on the present results and test specific independent variables as described. This is now included as one of the limitations of this study.

**6.** Lastly, I urge you to search for recently published articles about Covid-19 vaccine hesitancy among MENA -- there might have been some publications since the submission of your manuscript. **Response:** This is an excellent suggestion. Since submission of this manuscript, several studies have been published in this area. We have now cited most recent studies which were conducted among Arab Americans and included in the discussion section.

**Reviewer #2**: In the current study, authors have investigated the acceptance of COVID-19 vaccination in a Sample of Middle Eastern and North African in Texas, Houston area. The manuscript is well-written and informative. I recommend acceptance of the manuscript. I have minor comments as indicated below:

In the Introduction line 59, Reference 44 is out of order. It should be labelled as Ref 3. Same applies to Ref 44 line 102, Ref 48 line 138. **Response:** We apologize for this error. Reference order is now adjusted as recommended

Referencing issues are also noted in lines: 249, 231, 247 **Response:** Thank you for pointing out the error. References order is adjusted as recommended

In the Introduction line 77, replace United State with US **Response:** Done as recommended.

In the Introduction line 87-88, MENA should be defined in the first use **Response:** Done as recommended.

In the Methods, ethical approval is repeated twice in line 122 and in Line 129-132.
Line 192: change “Obesity” to “obesity”. **Response:** Thank you for pointing out these errors. Repeated ethical approval paragraph was deleted. The uppercase “O” in Obesity was replaced with lowercase “o”.

In the Discussion, I suggest that authors to compare their results to some Data from MENA countries. For Example: PMID: 36394768, PMID: 34014128 and PMID: 35857813 **Response:** Following suggestions of the reviewer, studies conducted in this area of research in MENA countries are now included and discussed in the revised version of the manuscript.
